# Organ-Specific Clinical Patterns of Pediatric Cystic Echinococcosis: A Multicenter Cohort from an Endemic Region

**DOI:** 10.3390/tropicalmed11100283

**Published:** 2026-10-08

**Authors:** Coskun Ekemen, Asli Arslan, Melis Deniz, Sema Yildirim Arslan, Nezihe Bilge Bahçeci, Ayşe Kübra Açık, Damla Seyhanlı, Emine Cigdem Ozer, Osman Uzunlu, Ülgen Çeltik, Nursen Belet, Dicle Sener Okur, Tuğçe Tural Kara, Ilknur Caglar, Derya Dirim Erdogan, Celal Çınar, Ülküm Zafer Dökümcü, Ahmet Çelik, Metin Korkmaz, Gulhadiye Avcu, Zumrut Sahbudak Bal

**Affiliations:** 1 Division of Infectious Diseases, Department of Pediatrics, Medical School of Ege University, 35100 Izmir, Turkey; coskunekemen@hotmail.com (C.E.); asly_k@hotmail.com (A.A.); semayildirimarslan@gmail.com (S.Y.A.); mdcigdemozer@gmail.com (E.C.O.); gul_akbas@yahoo.com.tr (G.A.); 2 Division of Infectious Diseases, Department of Pediatrics, Balıkesir Atatürk City Hospital, 10100 Balikesir, Turkey; mlsdnz@gmail.com; 3 Division of Infectious Diseases, Department of Pediatrics, Faculty of Medicine, Aydin Adnan Menderes University, 09100 Aydin, Turkey; nbilge17@hotmail.com (N.B.B.); ilknur.caglar@adu.edu.tr (I.C.); 4 Division of Infectious Diseases, Department of Pediatrics, Faculty of Medicine, Akdeniz University, 07070 Antalya, Turkey; aysekubra@akdeniz.edu.tr (A.K.A.); tugcekara@akdeniz.edu.tr (T.T.K.); 5 Division of Infectious Diseases, Department of Pediatrics, Faculty of Medicine, Dokuz Eylul University, 35340 Izmir, Turkey; damla.seyhanli@deu.edu.tr (D.S.); nurbelet@yahoo.com (N.B.); 6 Department of Pediatric Surgery, Faculty of Medicine, Pamukkale University, 20160 Denizli, Turkey; ouzunlu@pau.edu.tr; 7 Department of Pediatric Surgery, Medical School of Ege University, 35100 Izmir, Turkey; ulgenceltik1988@gmail.com (Ü.Ç.); zdokumcu@gmail.com (Ü.Z.D.); celikahmet969@gmail.com (A.Ç.); 8 Division of Infectious Diseases, Department of Pediatrics, Faculty of Medicine, Pamukkale University, 20160 Denizli, Turkey; dokur@pau.edu.tr; 9 Department of Medical Parasitology, Medical School of Ege University, 35100 Izmir, Turkey; derya.dirim.erdogan@ege.edu.tr (D.D.E.); metin.korkmaz@ege.edu.tr (M.K.); 10 Division of Cardiovascular and Interventional Radiology, Department of Radiology, Medical School of Ege University, 35100 Izmir, Turkey; celalcinar@hotmail.com

**Keywords:** cystic echinococcosis, pediatric hydatid disease, pulmonary involvement, albendazole, PAIR, multiorgan involvement

## Abstract

Pediatric cystic echinococcosis (CE) remains insufficiently characterized, particularly regarding organ-specific presentation and outcomes. This multicenter retrospective cohort included children aged 0–18 years with a definitive diagnosis of CE between 2013 and 2023 at six tertiary centers in Turkey. Among 119 patients (median age 10.6 years; 50.4% male), the liver was involved in 72.3% and the lungs in 36.1%, and 20.2% had two or more organs involved. Pulmonary involvement was associated with fever, higher C-reactive protein and complicated cysts (65.1% vs. 5.3%, *p* < 0.001). In multivariable analysis, fever (adjusted odds ratio 5.58, 95% confidence interval 1.85–16.81) and C-reactive protein (adjusted odds ratio 1.13 per 10 mg/L, 95% confidence interval 1.01–1.27) were associated with pulmonary involvement, with consistent results after multiple imputation and adjustment for center. Among patients with pulmonary involvement, fever was more frequent with complicated than with uncomplicated cysts (67.9% vs. 20.0%, *p* = 0.004), consistent with the recognized propensity of pulmonary cysts to rupture or become secondarily infected; fever in pulmonary CE may therefore primarily signal cyst complication. Hepatic CE was managed with albendazole alone, percutaneous treatment, surgery or watch-and-wait, and pulmonary CE mainly surgically (79.1%). During follow-up, post-index detection of CE was more frequent in patients with multiorgan involvement, including after accounting for follow-up duration. Pulmonary CE showed a more inflammatory and frequently complicated profile; fever in a child with pulmonary CE should prompt evaluation for cyst rupture or secondary infection.

## 1. Introduction

Cystic echinococcosis (CE) is a zoonotic parasitic disease caused by the larval stage of Echinococcus granulosus sensu lato. The parasite circulates between definitive hosts, mainly dogs and other canids, and intermediate hosts such as sheep and livestock, while humans become accidental intermediate hosts through ingestion of parasite eggs contaminating food, water, or soil [1].

CE remains endemic in many parts of the world, including South America, North Africa, the Mediterranean basin, Eastern Europe, the Middle East, and Central Asia. In these regions, the disease continues to impose substantial health and economic burdens, particularly in rural communities where close contact between livestock and dogs facilitates parasite transmission [1,2].

After ingestion, oncospheres penetrate the intestinal mucosa and enter the portal circulation. The liver is the most frequently affected organ, followed by the lungs, although cysts may occur in almost any tissue. While hepatic cysts constitute the majority of pediatric CE cases, pulmonary involvement requires careful clinical assessment because pulmonary cysts may grow rapidly and are more prone to bronchial communication, pleural rupture, and secondary infection. These complications may contribute to respiratory symptoms and elevated inflammatory markers. Therefore, accurate assessment of organ involvement, cyst morphology, and complications is important for clinical evaluation and treatment planning [1,3].

Imaging plays a central role in the diagnosis and management of CE. Early ultrasound-based classification of hydatid cysts was first described by Gharbi and colleagues, who categorized cyst morphology into five sonographic types reflecting different stages of cyst evolution [4]. Building on these morphological principles, the World Health Organization Informal Working Group on Echinococcosis (WHO-IWGE) later developed a standardized ultrasound classification system that categorizes cysts as active (CE1–CE2), transitional (CE3a–CE3b), and inactive (CE4–CE5), which is widely used to guide treatment decisions and follow-up strategies [1,5].

These imaging-based classifications are clinically important because treatment selection is closely linked to cyst stage and clinical context. Management is therefore determined not only by the available therapeutic options, but also by cyst stage, size, organ location, symptoms, complications, and local expertise. For hepatic CE, active or transitional cysts may require antiparasitic therapy, percutaneous intervention, or surgery depending on stage and size, whereas inactive uncomplicated cysts may be followed with a watch-and-wait approach in selected cases. In extrahepatic disease, treatment decisions require additional consideration of organ location and complications because the hepatic stage-specific framework may not apply uniformly to all sites [1,3,6].

Despite advances in diagnosis and treatment, evidence guiding management remains limited, particularly in pediatric populations where data on clinical characteristics and treatment outcomes are scarce [7,8,9]. Therefore, we conducted this multicenter study to characterize the clinical spectrum, organ distribution, hepatic cyst-stage distribution, recorded management, and follow-up findings of pediatric CE in an endemic region.

## 2. Materials and Methods

### 2.1. Study Design and Population

This multicenter retrospective cohort study included pediatric patients (0–18 years) diagnosed with CE between September 2013 and September 2023 across six tertiary care centers in Turkey. The study was conducted in accordance with the principles of the Declaration of Helsinki. Ethical approval was obtained from the Ethics Committee of Ege University Faculty of Medicine (Approval No: 23-12.1T/4).

At each center, all consecutive patients aged 0–18 years with a definitive diagnosis of CE during the study period were included. Patients were identified from pediatric infectious diseases, pediatric surgery and radiology/interventional radiology records and from hospital archives using diagnostic labels and record entries related to “cystic echinococcosis,” “hydatid cyst,” “Echinococcus,” and organ-specific hydatid disease. For each patient, clinical notes, imaging reports, laboratory records, operative or interventional procedure notes, pathology reports, treatment records and follow-up documentation were reviewed to confirm eligibility and extract study variables. Only patients meeting the definitive-diagnosis criteria described below were entered into the study database; no patient was excluded after entry, and the number of records screened but not meeting these criteria was not logged. The coordinating center contributed about one third of the patients (39/119); the distribution by center is shown in Supplementary Figure S1.

### 2.2. Diagnostic Criteria

Patients with a diagnosis of CE were eligible for inclusion. CE was required to be documented by characteristic imaging findings on ultrasonography, computed tomography, or magnetic resonance imaging, supported by serological testing when available. In patients who underwent surgery or interventional procedures, histopathological or parasitological confirmation was accepted as definitive diagnostic evidence when available. Clinical findings and serological positivity without compatible imaging or histopathological/parasitological confirmation were not considered sufficient for inclusion.

Supportive clinical findings and modes of presentation included abdominal pain, abdominal distension, nausea or vomiting, cough, chest pain, dyspnea, fever, hemoptysis, salty sputum expectoration, headache, or incidental/asymptomatic detection during imaging. Compatible imaging findings included cystic lesions interpreted as CE by radiologists and/or treating clinicians, including unilocular or multivesicular cysts, daughter cysts, detached or floating membranes, water-lily sign, calcified or inactive cyst morphology, or organ-specific cystic lesions consistent with hydatid disease.

Serological evaluation included enzyme-linked immunosorbent assay (ELISA) and indirect hemagglutination assay (IHA) when available. Histopathological confirmation was considered diagnostic when surgical specimens were obtained. Patients without definitive diagnostic evidence or with insufficient clinical data were excluded.

### 2.3. Clinical, Laboratory and Imaging Variables

Data were retrospectively extracted from the available medical records of each participating center using a standardized data extraction form. Collected patient-level variables included age, sex, place of residence, animal contact, family history of hydatid disease, presenting symptoms, hospitalization status, length of hospital stay, treatment modality, albendazole duration, follow-up duration, post-index detection of CE during follow-up, and treatment-related adverse events. Presenting symptoms, including fever, refer to findings documented at presentation (day of admission or first outpatient evaluation) and before any surgical or percutaneous procedure. Laboratory variables included CRP, ESR, leukocyte count, eosinophil count and percentage, ELISA IgG, IHA, and total IgE when available. Eosinophilia was defined as an absolute eosinophil count ≥500 cells/µL at diagnosis. Cyst-level and imaging variables included involved organ or organs, number of cysts, largest cyst diameter, hepatic lobe involvement, Gharbi stage, presence of multiple cysts, and complications such as rupture or infection. Multiorgan involvement was defined as the involvement of two or more organs on the basis of organ-level imaging data. A cyst was classified as complicated when infection, rupture (into the bronchial tree, pleural or peritoneal cavity or biliary tree) or peri-cystic infection was documented on imaging or at operation, in keeping with the definition of complicated CE used in current treatment frameworks [6]; complication status was assigned from these findings, independently of symptoms, fever or laboratory values. Treatment variables included medical therapy, PAIR or modified catheterization technique, surgery, and watch-and-wait management.

Imaging evaluation included ultrasonography, computed tomography, and magnetic resonance imaging when clinically indicated. Data regarding cyst location, number, size, and complications such as rupture or infection were recorded.

### 2.4. Cyst Staging

Hepatic cysts were staged according to the Gharbi ultrasound classification as recorded in the original imaging reports. In patients with multiple hepatic cysts, the stage of the most active cyst was recorded; when all cysts were of the same stage, the largest cyst was reported. Original ultrasound images were not re-reviewed and no reclassification into the WHO-IWGE system was attempted. For orientation, the approximate WHO-IWGE correspondence of each Gharbi type is indicated in parentheses (Type I, CE1; Type II, CE3a; Type III, CE2 or CE3b; Type IV, CE4; Type V, CE5) [1,4,5]; because Gharbi Type III encompasses both CE2 and CE3b, these categories are not separated.

### 2.5. Treatment

Treatment modalities were recorded as documented in the medical records. Treatment decisions were made by the treating clinicians according to organ involvement, cyst characteristics, cyst size, imaging-based stage or morphology, clinical presentation, complications, local expertise, and guideline recommendations available during the study period [1,4,5]. No study-specific treatment algorithm was applied. Recorded management modalities included medical therapy with albendazole (10–15 mg/kg/day in two divided doses), percutaneous interventions such as PAIR (puncture–aspiration–injection–reaspiration) or modified catheterization technique (Mo-CAT), surgical treatment, and watch-and-wait management for selected inactive cysts.

For hepatic CE, treatment modality was classified according to the first liver-directed management of the index hepatic cyst using a mutually exclusive hierarchy: (1) surgery, when open or laparoscopic surgery was the initial liver-directed procedure; (2) PAIR/Mo-CAT, when a percutaneous procedure was the initial liver-directed treatment, irrespective of subsequent surgery; (3) albendazole alone, when no liver-directed procedure was performed and the cyst was active or transitional (Gharbi I–III) or unstaged; and (4) watch-and-wait, when no liver-directed procedure was performed and the cyst was inactive (Gharbi IV–V). Surgery or percutaneous treatment of extrahepatic lesions did not alter the hepatic classification. Because the type of percutaneous procedure was recorded as a single variable, PAIR and Mo-CAT are reported together. For pulmonary CE, management was recorded as surgery, percutaneous procedure (any site) or albendazole alone.

### 2.6. Follow-Up Outcome

The follow-up outcome was defined as post-index detection of CE: the radiological documentation, after completion of the index treatment (the first treatment applied to the disease present at diagnosis), of an active cyst at the treated site or of a cyst not previously documented. According to the 2025 WHO terminology, true recurrence refers to the appearance of an active cyst at the site of a previously treated lesion, whereas reactivation refers to the progression of a previously inactive cyst; reinfection, secondary dissemination and previously undetected lesions are further distinct entities [1]. Because the anatomical relation of the subsequent lesion to the treated site was not consistently recorded, these could not be differentiated; post-index detection is therefore a composite outcome and is not interpreted as a recurrence endpoint. Follow-up imaging was performed at the discretion of the treating clinicians; the modality, number and intervals of follow-up imaging studies were not standardized and were not systematically recorded. Patients without post-treatment follow-up documented at the participating center (lost to follow-up or followed elsewhere) were not evaluable for this outcome and are reported separately. Where the date of the imaging study on which the lesion was first noted was documented, the interval from completion of index treatment to detection was recorded.

### 2.7. Statistical Analysis

Statistical analysis was performed using SPSS version 25.0 (IBM Corp., Armonk, NY, USA). Continuous variables were expressed as mean ± standard deviation (SD) or median [interquartile range (IQR)] depending on data distribution. Comparisons between groups were performed using the Mann–Whitney U test for continuous variables. Categorical variables were compared using the chi-square test or Fisher’s exact test, as appropriate. Candidate variables for the multivariable logistic regression model of pulmonary involvement were those associated with pulmonary involvement in univariable analysis (male sex, rural residence, fever, multiorgan involvement, complicated cyst, CRP, leukocyte count and IHA titer) together with age as an a priori confounder. Respiratory symptoms, hospitalization and surgery were not considered because they are consequences of pulmonary localization; multiorgan involvement was not considered because it is definitionally linked to the outcome; complicated cyst status was not considered because cyst complication, in particular bronchial rupture, is itself a manifestation of pulmonary localization, so that adjustment for it would constitute overadjustment; leukocyte count was omitted in favor of the collinear CRP; and IHA titer was omitted because of missingness. Given 37 pulmonary cases among the 83 patients with complete data, the primary model was limited to three pre-specified variables (rural residence, fever, and CRP per 10 mg/L increase; approximately 12 events per variable), entered simultaneously (Enter method) to avoid the instability of data-driven stepwise selection. Male sex, age and center (coordinating vs. other centers) were examined by adding them to the model in sensitivity analyses, and a model without CRP (rural residence and fever) was fitted in all 119 patients (Supplementary Table S2). Because CRP was missing in 36 patients, the primary model was also fitted after multiple imputation of CRP by chained equations with predictive mean matching (40 imputed datasets; imputation model including pulmonary involvement, rural residence, fever, sex, age, hospitalization, multiorgan involvement, leukocyte count and center), and estimates were combined using Rubin’s rules. Within patients with pulmonary involvement, fever and CRP were compared between complicated and uncomplicated cysts. Model fit is summarized by the likelihood-ratio χ^2^ and Nagelkerke R^2^, a pseudo-R^2^ rescaled to 0–1 that approximates the proportion of outcome variation explained by the model and is not interpretable as a linear-regression R^2^. A *p*-value < 0.05 was considered statistically significant. Apart from the multiple imputation analysis, missing data were not imputed; the number of patients with available data is given for every variable in the tables, and patients included in and excluded from the complete-case analysis were compared (Supplementary Table S2). The follow-up outcome was compared between patients with and without events among those with documented follow-up using univariable tests; to account for differences in follow-up duration, the event rate was also expressed per 100 person-years, the comparison was repeated among patients followed for at least 2 years, and associations with baseline features were additionally adjusted for follow-up duration by logistic regression. Because event timing was unavailable for some events and follow-up duration was recorded in whole years, no survival analysis was performed; the interval to detection is summarized as a median among event cases with timing data.

## 3. Results

### 3.1. Baseline Characteristics

A total of 119 children were included (median age 10.6 years; 50.4% male). About half lived in rural areas, and animal contact was reported for 59.0% of those with available information; a family history of hydatid disease was uncommon. Abdominal pain, cough and nausea–vomiting were the most frequent presenting symptoms, and 14 patients (11.8%) were asymptomatic (Table 1).

Most patients were hospitalized. Albendazole was temporarily discontinued in two patients because of elevated liver enzymes. Post-treatment follow-up was documented for 104 patients (87.4%); the remaining 15 were not evaluable for the follow-up outcome, which is described in Section 3.5.

**Figure 1:**
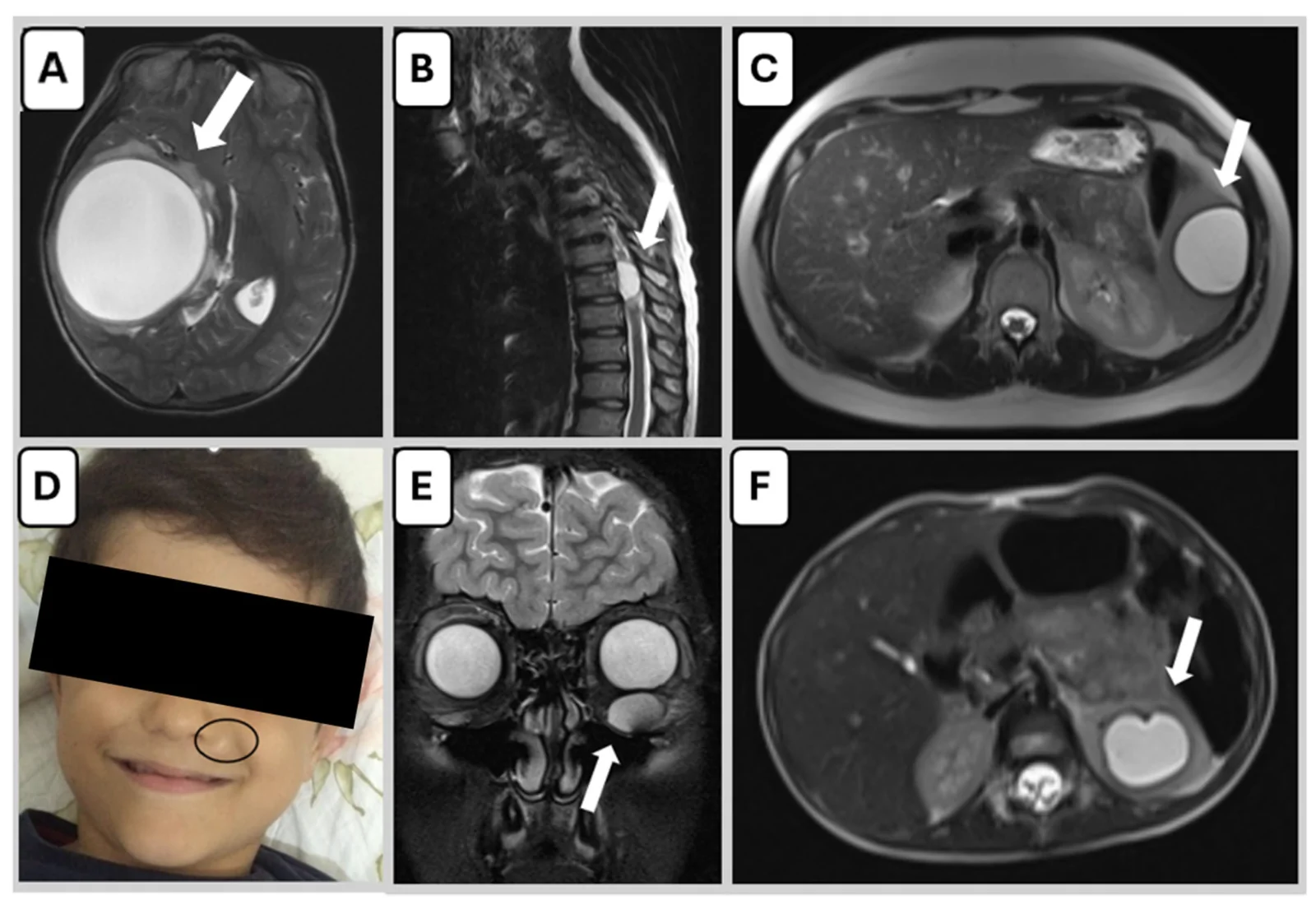
Composite MRI and clinical images demonstrating rare organ involvement in pediatric cystic echinococcosis. (**A**) Axial T2-weighted/fluid-sensitive brain MRI showing a large cerebral hydatid cyst. (**B**) Sagittal T2-weighted/fluid-sensitive spinal MRI demonstrating a cystic lesion causing spinal cord compression. (**C**) Axial T2-weighted abdominal MRI showing a splenic hydatid cyst. (**D**) Clinical image of a cutaneous hydatid cyst located on the upper lip region. (**E**) Coronal T2-weighted/fluid-sensitive orbital MRI illustrating a cyst within the inferior rectus muscle of the left orbit. (**F**) Axial fat-suppressed T2-weighted/fluid-sensitive abdominal MRI showing a well-defined cystic lesion in the left kidney with a thin internal membrane, consistent with a hydatid cyst. Arrows indicate the cysts; the ellipse in (**D**) marks the cutaneous cyst.

The liver was involved in 86 patients (72.3%) and the lungs in 43 (36.1%); 19 (16.0%) had concomitant liver and lung disease, and 24 (20.2%) had two or more organs involved (Table 2). Representative examples of rare localizations are shown in Figure 1.

**Figure 2:**
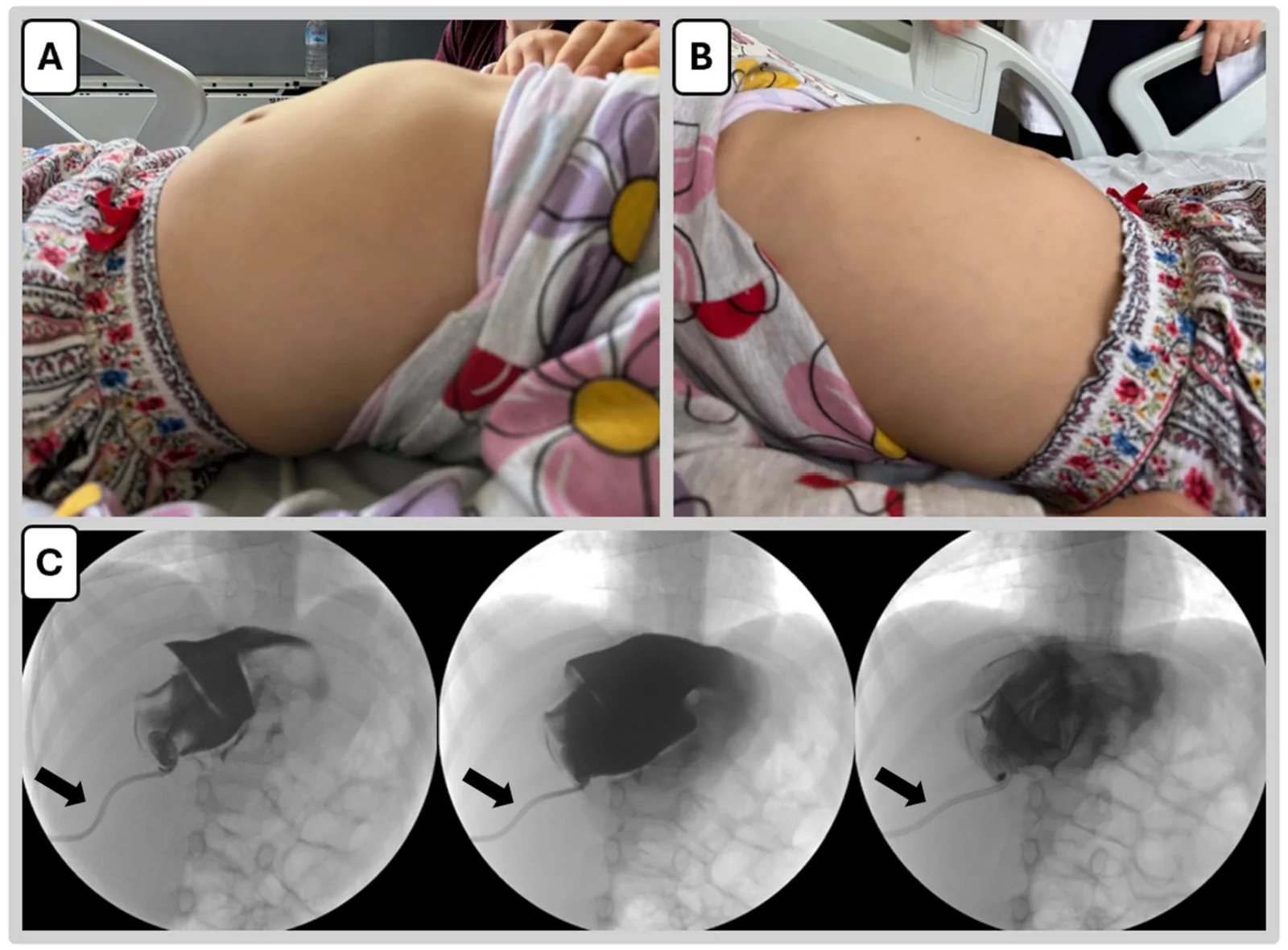
Clinical and procedural presentation of a pediatric patient with hepatic cystic echinococcosis. (**A**,**B**) Externally visible right upper quadrant bulging due to a large hepatic hydatid cyst in a 5-year-old girl with no prior medical history and documented contact with domestic animals. (**C**) Sequential fluoroscopic images obtained during the modified catheterization technique (Mo-CAT). Under ultrasound guidance, the cyst was punctured with an 18G needle and its clear content aspirated; an 8F drainage catheter was placed over a guidewire, the cyst was completely evacuated, hypertonic saline equal to 30–40% of the aspirated volume was injected and re-aspirated after 15 min, and the catheter was left in place for free drainage. Images obtained by contrast injection through the catheter show the opacified cyst cavity and its progressive collapse; the catheter is indicated by the arrows. A biliary fistula developed after the procedure and required surgical repair.

Multiple cysts were present in 37.8% of patients and complicated cysts in 26.9%; the largest cyst exceeded 5 cm in nearly two thirds. In rare cases, large hepatic cysts produced visible right upper quadrant bulging on physical examination (Figure 2A,B). Laboratory findings, including eosinophilia (≥500 cells/µL) in 29.4%, are summarized in Table 2.

### 3.2. Factors Associated with Pulmonary Involvement

Patients with pulmonary involvement were more often male and of rural residence, were all hospitalized, and more frequently presented with fever and respiratory symptoms (Table 3). Multiorgan involvement (46.5% vs. 5.3%) and complicated cysts (65.1% vs. 5.3%) were markedly more common, and CRP, leukocyte counts and IHA titers were higher, whereas age, the proportion with multiple cysts, cyst size, eosinophil counts and ELISA titers did not differ between groups. Surgery at any site was performed in 79.1% of patients with pulmonary involvement compared with 44.7% of those without.

Multivariable logistic regression was performed in the 83 patients with complete data for the three pre-specified variables, 37 of whom had pulmonary involvement (Table 4). Fever (adjusted OR 5.58, 95% CI 1.85–16.81) and CRP (adjusted OR 1.13 per 10 mg/L increase, 95% CI 1.01–1.27) were associated with pulmonary involvement, whereas rural residence was not (adjusted OR 1.71, 95% CI 0.59–4.90). In further sensitivity analyses (Supplementary Table S2), the estimate for fever was similar after adding male sex (adjusted OR 5.02, 95% CI 1.63–15.45) or male sex and age (adjusted OR 4.86, 95% CI 1.56–15.11), whereas the estimate for CRP was attenuated and no longer statistically significant (adjusted OR 1.12, 95% CI 0.99–1.26 and 1.00–1.26, respectively); male sex was not associated with pulmonary involvement in any model. Adding center did not materially change the estimates (fever: adjusted OR 5.45, 95% CI 1.79–16.59; CRP: adjusted OR 1.13, 95% CI 1.01–1.28; coordinating center: adjusted OR 1.17, 95% CI 0.39–3.53), although the proportion with pulmonary involvement differed across centers (*p* = 0.013). In a model without CRP fitted in all 119 patients, fever remained associated with pulmonary involvement (adjusted OR 6.10, 95% CI 2.45–15.17). The 36 patients excluded from the complete-case analysis because CRP was not available had less pulmonary involvement, fever and multiorgan disease than those included (Supplementary Table S2). All 36 came from three centers in which CRP was not routinely recorded (available for 10–58% of their patients), whereas CRP was available for all patients from the other three centers. After multiple imputation of missing CRP values, the results were consistent with the complete-case analysis (fever: adjusted OR 4.35, 95% CI 1.65–11.49; CRP: adjusted OR 1.14, 95% CI 1.01–1.27; Table 4).

### 3.3. Pulmonary Cystic Echinococcosis

Of the 43 patients with pulmonary involvement, 24 had lung-only disease and 19 had concomitant hepatic disease (Table 5). A complicated cyst was recorded in 18 of the 24 lung-only patients and in 10 of the 19 with combined disease, whereas multiple cysts were recorded in 2 and 16 patients, respectively. Fever was recorded in 19 of 28 patients (67.9%) with a complicated pulmonary cyst and in 3 of 15 (20.0%) with an uncomplicated pulmonary cyst (*p* = 0.004); median CRP was 30 vs. 10.2 mg/L (*p* = 0.37). Surgery was performed in 34 patients (79.1%), a percutaneous procedure at any site in 11, and six patients (14.0%) received albendazole alone; a treatment-related complication was recorded in three patients. Post-index detection of CE occurred in 11 of 37 patients with documented follow-up, in 2 of 21 with lung-only disease and 9 of 16 with combined liver and lung disease.

### 3.4. Recorded Management in Hepatic Cystic Echinococcosis

Among the 86 patients with hepatic involvement, first liver-directed management was albendazole alone in 30 (34.9%), PAIR/Mo-CAT in 31 (36.0%), surgery in 19 (22.1%) and watch-and-wait in 6 (7.0%) (Table 6); 12 patients treated with PAIR/Mo-CAT subsequently underwent surgery, including the patient with a post-procedural biliary fistula shown in Figure 2C. Cysts treated percutaneously were predominantly Gharbi Type I (64.5%), whereas Type III cysts were more frequent in the surgical group (35.3% vs. 9.7%), and all cysts managed by watch-and-wait were Type IV or V. Post-index detection of CE occurred in 10 of 31 patients after PAIR/Mo-CAT and in 3 of 17 after surgery.

### 3.5. Post-Index Detection of Cystic Echinococcosis During Follow-Up

Among the 104 patients with documented follow-up, post-index detection of CE was documented in 19 (18.3%); their individual characteristics are given in Supplementary Table S1. Seventeen had hepatic and eleven had pulmonary involvement at baseline, including 9 with both. Compared with patients without an event, those with post-index detection more often had multiorgan involvement (57.9% vs. 11.8%) and pulmonary involvement (57.9% vs. 30.6%) and had a higher number of cysts, whereas the proportions with multiple cysts, with a largest cyst >5 cm and with complicated cysts did not differ (Table 7). Documented follow-up was longer in event cases (median 3 vs. 2 years). The event rate was 5.7 per 100 person-years (19 events over 334 person-years), and 16 of 75 patients (21.3%) followed for at least 2 years had an event. The association with multiorgan involvement persisted after adjustment for follow-up duration (OR 9.27, 95% CI 2.94–29.24) and among patients followed for at least 2 years (9/17 vs. 7/58, *p* < 0.001); the association with pulmonary involvement was retained after adjustment for follow-up duration (OR 3.61, 95% CI 1.23–10.59) but was attenuated among patients followed for at least 2 years (9/26 vs. 7/49, *p* = 0.073). The date of detection was documented for 14 of the 19 events, with a median interval of 15.5 months (IQR 4.8–36) after index treatment; the five events without a documented date were similar to the others in follow-up duration and organ involvement (Supplementary Table S1).

## 4. Discussion

This multicenter cohort provides an integrated organ-specific characterization of pediatric CE across clinical presentation, inflammatory profile, management, and follow-up. Hepatic disease remained the predominant localization, but pulmonary involvement affected more than one third of patients and clustered with fever, respiratory symptoms, multiorgan disease, complicated cysts, and higher inflammatory markers. By evaluating these features within the same pediatric cohort, our study extends the available evidence beyond organ-frequency estimates and highlights the clinical consequences of pulmonary localization. This pattern is consistent with the organ-specific behavior of pulmonary cysts, including their tendency to enlarge, communicate with the bronchial tree, rupture, and become secondarily infected [8,10].

The liver was the most frequently involved organ in our cohort, affecting 72.3% of patients, followed by the lungs in 36.1%; concomitant liver and lung disease was present in 16.0%, and one fifth of patients had two or more organs involved. This distribution is consistent with the portal filtration mechanism, whereby the liver acts as the principal barrier for oncospheres entering the portal circulation and the lungs constitute the second major site of cyst development. Similar organ distributions have been reported in epidemiological studies and multicenter cohorts from endemic regions, including large datasets from Spain and other European countries [11,12,13,14]. In the population-based HERACLES ultrasound survey conducted in endemic rural areas of Bulgaria, Romania, and Turkey, abdominal CE was also predominantly hepatic, and active cysts were identified across different age groups, including children [14].

Nevertheless, pulmonary disease appears particularly prominent in pediatric CE. General descriptions of CE estimate pulmonary involvement at approximately 15–20% in overall populations [5,6], whereas 36.1% of the children in our cohort had pulmonary involvement. Some studies have similarly suggested that the lung-to-liver ratio may be higher in children than in adults [11]. The compressibility, vascularization, and negative pressure of the developing lung may permit more rapid cyst expansion and earlier clinical expression [8,11]. Pediatric cohorts from endemic regions have also identified pulmonary and multiorgan involvement as clinically important patterns, although hepatic disease generally remains the most frequent localization [8]. Thus, the conventional liver–lung hierarchy is retained in children, while pulmonary involvement represents a comparatively prominent and clinically consequential component of pediatric disease.

Pulmonary involvement was associated with a distinctly more symptomatic presentation. Fever and respiratory symptoms were more frequent, and complicated cysts were present in 65.1% of pulmonary cases compared with 5.3% of non-pulmonary cases. This finding is consistent with the ability of pulmonary cysts to expand within elastic lung tissue and subsequently rupture into the bronchial tree or pleural cavity, producing acute respiratory manifestations and facilitating secondary infection [9,15]. CRP levels and leukocyte counts were also higher among patients with pulmonary involvement. Fever and CRP remained associated with pulmonary involvement in the multivariable model, including after multiple imputation of missing CRP values and adjustment for center. Complicated cyst status was not entered as a covariate, because cyst complication, in particular bronchial rupture, is itself a manifestation of pulmonary localization. Within patients with pulmonary involvement, however, fever was considerably more frequent in those with complicated cysts, whereas CRP was numerically but not significantly higher, possibly reflecting the small number of uncomplicated pulmonary cysts. This pattern is consistent with clinical series in which ruptured or complicated pulmonary cysts presented with fever, expectoration and hemoptysis [15,16] and in which children frequently presented only after cyst rupture [17,18], and with the recognized propensity of pulmonary cysts to rupture into the bronchial tree or pleura and to become secondarily infected [1,5,18]. Fever in a child with pulmonary CE should therefore prompt evaluation for cyst rupture or secondary infection. These concurrently measured findings describe the clinical profile of pulmonary CE and do not establish temporal or causal relationships [3,19]. Missing CRP values were confined to three centers in which CRP was not routinely recorded, and the results after multiple imputation were consistent with the complete-case analysis.

These organ-specific differences have direct implications for management. Surgery remains the principal treatment for operable pulmonary hydatid cysts in children, including both uncomplicated and complicated disease [15,20], and was performed in 79.1% of the pulmonary cases in this cohort. Hepatic CE, by contrast, is generally managed within an imaging-based framework incorporating cyst stage or morphology, size, symptoms, complications, and local expertise. Depending on these factors, management may include albendazole therapy, percutaneous intervention, surgery, or watch-and-wait follow-up [3,6,21]. The findings of the present study reinforce the importance of adapting clinical evaluation and treatment planning to the involved organ rather than applying a uniform management approach across disease sites.

The hepatic treatment findings further illustrate the importance of clinical and cyst-stage context. Among patients with recorded Gharbi stage, Type I cysts predominated in the PAIR/Mo-CAT group, whereas Type III cysts were more frequent in the surgical group, and all cysts managed by watch-and-wait were inactive (Type IV–V). These distributions likely reflect clinical selection according to cyst morphology, size, complications, organ involvement, local expertise, and clinician judgment [1,22]. Because treatment allocation was not standardized and the treatment groups differed in their baseline clinical and cyst-stage characteristics, the findings by treatment group, including the proportions with post-index detection of CE, are descriptive and cannot establish comparative treatment effectiveness.

Albendazole therapy was generally well tolerated. Temporary discontinuation was required in only two patients because of elevated liver enzyme levels. This finding is consistent with previous pediatric studies indicating that albendazole is usually well tolerated, although periodic liver function monitoring remains necessary during treatment [3,23,24].

Post-index detection of CE occurred in 18.3% of patients with documented follow-up (5.7 per 100 person-years). This composite outcome combines true recurrence, reactivation, reinfection, secondary dissemination and previously undetected lesions, which could not be distinguished in the retrospective records; it is therefore not comparable with recurrence rates from studies applying WHO-defined recurrence and should not be read as such. Events were concentrated among patients with multiorgan involvement, an association that persisted after accounting for follow-up duration, whereas the association with pulmonary involvement was less robust and cyst size was not associated with the outcome. Follow-up was not standardized, and the longer documented follow-up of event cases probably reflects both closer surveillance of patients with extensive disease and continued follow-up after a new lesion was detected. With these caveats, the findings are in line with previous pediatric studies linking disease extent with post-treatment events [8,10,25]; environmental exposure and host factors may additionally influence ongoing transmission in endemic regions [26,27], and surgical data associate multisite disease with adverse postoperative outcomes [28].

The principal strengths of this study are its multicenter design, relatively large pediatric cohort, and integrated assessment of hepatic, pulmonary, and less common organ involvement. The availability of detailed clinical, laboratory, imaging, treatment, and follow-up data enabled organ-specific patterns to be examined within the same population. Inclusion of six tertiary centers also captured variation in clinical presentation and real-world management across referral settings in an endemic region.

Several limitations should be considered. The retrospective design and tertiary referral setting may have introduced selection bias towards symptomatic or complicated disease; about one third of the cohort came from the coordinating center, and the number of records screened but not meeting the diagnostic criteria was not logged. Clinical assessment, imaging documentation, treatment selection and follow-up practices varied across centers and over the 10-year period; adjustment for center did not change the main estimates, but residual center-related confounding cannot be excluded, and the small number of centers precluded mixed-effects modelling. Laboratory data were incomplete (CRP available in 83, ESR in 51 and ELISA IgG in 48 patients); missing CRP reflected center documentation practice, and multiple imputation, which gave consistent results, assumes that missingness depends only on observed variables. Fever and CRP were recorded at presentation, concurrently with complication status; the study was designed to characterize the clinical profile of pulmonary CE rather than to estimate the independent contribution of each factor. Albendazole duration was not retrievable for 14 patients. Treatment allocation was non-randomized, PAIR and Mo-CAT could not be separated, and heterogeneity in surgical approaches precluded subgroup analyses, so treatment groups are described only. The follow-up outcome is a composite whose components could not be differentiated; follow-up was documented in 87% of patients, imaging schedules were not standardized, follow-up was longer in patients with events, the date of detection was unavailable for five events, and no survival analysis was possible. Finally, hepatic staging relied on the Gharbi classification recorded in the original reports; images were not re-reviewed and WHO-IWGE stages are given only as approximate correspondences, so Gharbi Type III cysts could not be assigned to CE2 or CE3b [1,4,5]. Broader routine use of the WHO-IWGE classification, standardized follow-up schedules and WHO-consistent outcome definitions would improve comparability between centers, and prospective pediatric studies incorporating these elements are needed to clarify organ-specific trajectories, post-treatment outcomes and the clinical implications of disease extent.

## 5. Conclusions

In this multicenter pediatric cohort from an endemic region, CE showed marked organ-specific clinical heterogeneity. Although the liver remained the most frequently involved organ, pulmonary disease affected more than one third of patients and was characterized by more frequent respiratory symptoms, complicated cysts and higher inflammatory markers. Consistent with the propensity of pulmonary cysts to rupture and become secondarily infected, fever was more frequent with complicated pulmonary cysts, and fever in a child with pulmonary CE should prompt evaluation for cyst complication. These findings support organ-adapted clinical assessment and management, with particular attention to pulmonary complications and multiorgan involvement. Albendazole was generally well tolerated. Post-index detection of CE during follow-up, most frequent in patients with multiorgan disease, underlines the need for structured follow-up, the optimal schedule of which cannot be defined from these data. Prospective multicenter studies using standardized WHO-IWGE staging, uniform follow-up protocols and harmonized outcome definitions are needed to clarify organ-specific trajectories and post-treatment outcomes in pediatric CE.

## Figures and Tables

**Table 1:** Baseline characteristics, treatment and outcomes of the study population (*n* = 119).

Variable	Total (*n* = 119), *n* (%) or Median [IQR]	*n* with Data
**Demographic characteristics**		
Age at diagnosis (years), median [IQR]	10.6 [8.2–13.8]	119
Male sex, *n* (%)	60 (50.4)	119
Rural residence, *n* (%)	58 (48.7)	119
Animal contact, *n* (%)	49 (59.0)	83
Family history of hydatid disease, *n* (%)	6 (5.0)	119
**Clinical presentation** **, *n* (%)**		
Abdominal pain	68 (57.1)	119
Cough	40 (33.6)	119
Nausea and vomiting	34 (28.6)	119
Fever	32 (26.9)	119
Chest pain	21 (17.6)	119
Flank pain	20 (16.8)	119
Dyspnea	19 (16.0)	119
Abdominal distension	16 (13.4)	119
Salty sputum expectoration	13 (10.9)	119
Hemoptysis	5 (4.2)	119
Headache	3 (2.5)	119
Asymptomatic	14 (11.8)	119
**Treatment and outcomes**		
Hospitalization required, *n* (%)	98 (82.4)	119
Length of hospital stay (days), median [IQR]	7.5 [5–12]	96
Total albendazole duration (months), median [IQR]	6.5 [4–12]	90
Documented post-treatment follow-up, *n* (%)	104 (87.4)	119
Follow-up duration (years), median [IQR]	2 [1–4]	104
Post-index detection of CE, *n* (%)	19 (18.3)	104
Interval from index treatment to detection (months), median [IQR]	15.5 [4.8–36]	14

Section headings are shown in bold. Asymptomatic, no presenting symptom recorded. Post-index detection of CE is a composite outcome (true recurrence, reactivation, reinfection, secondary dissemination or previously undetected lesion) reported among patients with documented post-treatment follow-up; the interval to detection is the median among event cases with a documented date of detection. CE, cystic echinococcosis; IQR, interquartile range.

**Table 2:** Organ involvement, cyst characteristics and laboratory findings (*n* = 119).

Variable	Total (*n* = 119), *n* (%) or Median [IQR]	*n* with Data
**Organ involvement, *n* (%)**		
Liver	86 (72.3)	119
Lung	43 (36.1)	119
Spleen	5 (4.2)	119
Kidney	3 (2.5)	119
Cranial	2 (1.7)	119
Skin	1 (0.8)	119
Spinal	1 (0.8)	119
Bladder	1 (0.8)	119
Muscle	1 (0.8)	119
Liver and lung (concomitant)	19 (16.0)	119
Multiorgan involvement (≥2 organs)	24 (20.2)	119
**Cyst characteristics**		
Multiple cysts, *n* (%)	45 (37.8)	119
Complicated cyst, *n* (%)	32 (26.9)	119
Largest cyst diameter (mm), median [IQR]	60 [42.5–82.5]	117
Largest cyst ≤5 cm, *n* (%)	43 (36.1)	119
Largest cyst >5 cm, *n* (%)	76 (63.9)	119
**Laboratory findings**		
CRP (mg/L), median [IQR]	6 [2–55]	83
ESR (mm/h), median [IQR]	21 [12–35]	51
Leukocyte count (cells/µL), median [IQR]	9090 [7500–12,700]	119
Eosinophil count (cells/µL), median [IQR]	250 [110–680]	119
Eosinophil percentage (%), median [IQR]	3 [1.18–6.02]	86
Eosinophilia (≥500 cells/µL), *n* (%)	35 (29.4)	119
ELISA IgG titer, median [IQR]	1/1280 [1/640–1/5000]	48
IHA titer, median [IQR]	1/320 [1/320–1/2500]	82
Total IgE (IU/mL), median [IQR]	218 [116–419]	11

Section headings are shown in bold. Multiorgan involvement, two or more organs involved on imaging: liver + lung (19), liver + spleen (2), lung + cranial (1), liver + skin (1), liver + cranial (1). Complicated cyst, infection, rupture or peri-cystic infection documented on imaging or at operation. Eosinophilia, absolute eosinophil count ≥500 cells/µL at diagnosis. Titers are reciprocal dilutions. CRP, C-reactive protein; ESR, erythrocyte sedimentation rate; ELISA, enzyme-linked immunosorbent assay; IHA, indirect hemagglutination assay; IgE, immunoglobulin E; IQR, interquartile range.

**Table 3:** Clinical and laboratory features according to pulmonary involvement.

Variable	Pulmonary Involvement (*n* = 43)	*n*	No Pulmonary Involvement (*n* = 76)	*n*	*p* Value
**Demographic and clinical characteristics**					
Age (years), median [IQR]	9.9 [8–11.9]	43	11 [8.4–14.5]	76	0.142
Male sex, *n* (%)	28 (65.1)	43	32 (42.1)	76	0.016
Rural residence, *n* (%)	27 (62.8)	43	31 (40.8)	76	0.021
Hospitalization required, *n* (%)	43 (100.0)	43	55 (72.4)	76	<0.001
Length of hospital stay (days), median [IQR]	8.5 [6–15.5]	42	7 [3–11.2]	54	0.088
Fever, *n* (%)	22 (51.2)	43	10 (13.2)	76	<0.001
Cough, *n* (%)	39 (90.7)	43	1 (1.3)	76	<0.001
Chest pain, *n* (%)	21 (48.8)	43	0 (0.0)	76	<0.001
Dyspnea, *n* (%)	19 (44.2)	43	0 (0.0)	76	<0.001
Salty sputum expectoration, *n* (%)	13 (30.2)	43	0 (0.0)	76	<0.001
Hemoptysis, *n* (%)	5 (11.6)	43	0 (0.0)	76	0.005
Multiorgan involvement (≥2 organs), *n* (%)	20 (46.5)	43	4 (5.3)	76	<0.001
**Cyst characteristics**					
Multiple cysts, *n* (%)	18 (41.9)	43	27 (35.5)	76	0.494
Complicated cyst, *n* (%)	28 (65.1)	43	4 (5.3)	76	<0.001
Largest cyst diameter (mm), median [IQR]	63 [45.8–82]	42	60 [40–90]	75	0.581
Largest cyst >5 cm, *n* (%)	30 (69.8)	43	46 (60.5)	76	0.313
**Laboratory findings**					
CRP (mg/L), median [IQR]	17 [3–94]	37	3.4 [2–13.6]	46	0.008
ESR (mm/h), median [IQR]	23 [12–36]	25	20 [12–32]	26	0.799
Leukocyte count (cells/µL), median [IQR]	11,400 [8510–15,540]	43	8650 [6998–11,302]	76	0.003
Eosinophil count (cells/µL), median [IQR]	280 [110–680]	43	235 [102–658]	76	0.416
Eosinophilia (≥500 cells/µL), *n* (%)	14 (32.6)	43	21 (27.6)	76	0.571
ELISA IgG titer, median [IQR]	1/1280 [1/640–1/5000]	25	1/1200 [1/640–1/5000]	23	0.654
IHA titer, median [IQR]	1/640 [1/320–1/5000]	29	1/320 [1/205–1/1250]	53	0.034
**Treatment and outcomes**					
Total albendazole duration (months), median [IQR]	6 [4–14]	39	7 [4–10]	51	0.402
Surgery (any site), *n* (%)	34 (79.1)	43	34 (44.7)	76	<0.001
Follow-up duration (years), median [IQR]	2 [1–4]	37	2 [1–4]	67	0.571
Post-index detection of CE, *n* (%)	11 (29.7)	37	8 (11.9)	67	0.025

Section headings are shown in bold. Values are *n* (%) or median [IQR]; *n*, number of patients with available data. Chi-square test (Fisher’s exact test when any expected count <5) for categorical and Mann–Whitney U test for continuous variables. Surgery refers to any anatomical site. Complicated cyst, infection, rupture or peri-cystic infection documented on imaging or at operation. Post-index detection of CE is reported among patients with documented follow-up. CE, cystic echinococcosis; CRP, C-reactive protein; ESR, erythrocyte sedimentation rate; ELISA, enzyme-linked immunosorbent assay; IHA, indirect hemagglutination assay; IQR, interquartile range.

**Table 4:** Multivariable logistic regression for pulmonary involvement: complete-case analysis and analysis after multiple imputation of missing CRP values.

Variable	Complete-Case Analysis (*n* = 83): Adjusted OR (95% CI)	*p* Value	Multiple Imputation (*n* = 119): Adjusted OR (95% CI)	*p* Value
Rural residence	1.71 (0.59–4.90)	0.322	2.30 (0.92–5.73)	0.074
Fever	5.58 (1.85–16.81)	0.002	4.35 (1.65–11.49)	0.003
CRP (per 10 mg/L increase)	1.13 (1.01–1.27)	0.038	1.14 (1.01–1.27)	0.027

Values are adjusted odds ratios (OR) with 95% confidence intervals (CI). Variables were entered simultaneously (Enter method); the model contains three pre-specified variables (approximately 12 pulmonary cases per variable in the complete-case analysis). Complete-case model: χ^2^ = 24.16, *p* < 0.001; Nagelkerke R^2^ = 0.338 (a pseudo-R^2^ rescaled to 0–1 that approximates the proportion of outcome variation explained by the model and is not interpretable as a linear-regression R^2^). Multiple imputation: 40 datasets imputed by chained equations with predictive mean matching, including center; estimates combined using Rubin’s rules. Additional sensitivity models are given in Supplementary Table S2. CRP, C-reactive protein.

**Table 5:** Presentation, management, complications and follow-up of patients with pulmonary cystic echinococcosis (*n* = 43).

	All Pulmonary (*n* = 43)	Lung Only (*n* = 24)	Liver and Lung (*n* = 19)
**Clinical presentation**			
Fever, *n* (%)	22 (51.2)	16 (66.7)	6 (31.6)
Cough, *n* (%)	39 (90.7)	23 (95.8)	16 (84.2)
Dyspnea, *n* (%)	19 (44.2)	12 (50.0)	7 (36.8)
Chest pain, *n* (%)	21 (48.8)	12 (50.0)	9 (47.4)
Salty sputum expectoration, *n* (%)	13 (30.2)	8 (33.3)	5 (26.3)
Hemoptysis, *n* (%)	5 (11.6)	3 (12.5)	2 (10.5)
**Cyst characteristics and complications**			
Multiple cysts, *n* (%)	18 (41.9)	2 (8.3)	16 (84.2)
Largest cyst diameter (mm), median [IQR]	63 [45.8–82] [*n* = 42]	60 [44.2–74.5]	69 [53.8–84.8] [*n* = 18]
Largest cyst >5 cm, *n* (%)	30 (69.8)	15 (62.5)	15 (78.9)
Complicated cyst, *n* (%)	28 (65.1)	18 (75.0)	10 (52.6)
**Management**			
Surgery, *n* (%)	34 (79.1)	20 (83.3)	14 (73.7)
PAIR/Mo-CAT (any site), *n* (%)	11 (25.6)	2 (8.3)	9 (47.4)
Albendazole alone (no surgery, no percutaneous procedure), *n* (%)	6 (14.0)	4 (16.7)	2 (10.5)
Total albendazole duration (months), median [IQR]	6 [4–14] [*n* = 39]	6 [3.8–11.2] [*n* = 22]	13 [3.5–22.5] [*n* = 17]
Length of hospital stay (days), median [IQR]	8.5 [6–15.5] [*n* = 42]	11 [6–17] [*n* = 23]	8 [5–12]
Treatment-related complication recorded, *n* (%)	3 (7.0)	2 (8.3)	1 (5.3)
**Follow-up**			
Follow-up duration (years), median [IQR]	2 [1–4] [*n* = 37]	2 [1–4] [*n* = 21]	2 [2–5.2] [*n* = 16]
Post-index detection of CE, *n*/*N* (%)	11/37 (29.7)	2/21 (9.5)	9/16 (56.2)

Section headings are shown in bold. Values are *n* (%) or median [IQR]; where the number with available data differs from the column total, it is shown in brackets. Surgery and PAIR/Mo-CAT refer to any anatomical site; in patients with liver and lung disease the site of each procedure was not separately recorded. Albendazole alone, no surgical or percutaneous procedure at any site. Complicated cyst, infection, rupture or peri-cystic infection documented on imaging or at operation. All 43 patients were hospitalized. Post-index detection of CE is reported among patients with documented follow-up. CE, cystic echinococcosis; IQR, interquartile range; Mo-CAT, modified catheterization technique; PAIR, puncture–aspiration–injection–reaspiration.

**Table 6:** Patients with hepatic cystic echinococcosis according to first liver-directed management (*n* = 86).

	Total (*n* = 86)	Albendazole Alone (*n* = 30)	PAIR/Mo-CAT (*n* = 31)	Surgery (*n* = 19)	Watch-and-Wait (*n* = 6)
**Demographic and clinical characteristics**					
Age (years), median [IQR]	10.9 [8.3–14.2]	10.8 [8.4–13.9]	10.6 [6.5–12.9]	13.3 [9.3–14.6]	10.1 [7.6–13.9]
Male sex, *n* (%)	41 (47.7)	14 (46.7)	16 (51.6)	7 (36.8)	4 (66.7)
Concomitant pulmonary involvement, *n* (%)	19 (22.1)	8 (26.7)	9 (29.0)	0 (0.0)	2 (33.3)
Hospitalization required, *n* (%)	67 (77.9)	13 (43.3)	31 (100.0)	19 (100.0)	4 (66.7)
Length of hospital stay (days), median [IQR]	7 [4–12] [*n* = 66]	8 [2.5–12] [*n* = 13]	7 [4–11]	8.5 [5–12.2] [*n* = 18]	7 [3.2–23.5] [*n* = 4]
**Cyst characteristics**					
Right lobe involvement, *n* (%)	81 (94.2)	28 (93.3)	30 (96.8)	17 (89.5)	6 (100.0)
Left lobe involvement, *n* (%)	19 (22.1)	7 (23.3)	4 (12.9)	6 (31.6)	2 (33.3)
Multiple cysts, *n* (%)	43 (50.0)	16 (53.3)	14 (45.2)	10 (52.6)	3 (50.0)
Complicated cyst, *n* (%)	14 (16.3)	6 (20.0)	5 (16.1)	2 (10.5)	1 (16.7)
Largest cyst diameter (mm), median [IQR]	60.5 [42.2–90] [*n* = 84]	48.5 [29.2–70] [*n* = 28]	70 [55–92]	78 [55–105]	42.5 [37.2–58.2]
Largest cyst >5 cm, *n* (%)	57 (66.3)	15 (50.0)	26 (83.9)	14 (73.7)	2 (33.3)
**Gharbi stage, *n* (% of staged)**					
Type I	35 (43.8)	9 (34.6)	20 (64.5)	6 (35.3)	0 (0.0)
Type II	21 (26.2)	9 (34.6)	7 (22.6)	5 (29.4)	0 (0.0)
Type III	17 (21.2)	8 (30.8)	3 (9.7)	6 (35.3)	0 (0.0)
Type IV	2 (2.5)	0 (0.0)	1 (3.2)	0 (0.0)	1 (16.7)
Type V	5 (6.2)	0 (0.0)	0 (0.0)	0 (0.0)	5 (83.3)
Stage not documented, *n*	6	4	0	2	0
**Laboratory findings**					
CRP (mg/L), median [IQR]	8.1 [2–48.7] [*n* = 58]	4.3 [2–30] [*n* = 19]	10 [2–53] [*n* = 23]	10 [2–55] [*n* = 11]	8.4 [2.3–53.5] [*n* = 5]
ESR (mm/h), median [IQR]	21 [12–34] [*n* = 36]	18 [10–34] [*n* = 10]	22 [13–34] [*n* = 16]	28 [12–49] [*n* = 7]	28 [11–39] [*n* = 3]
Leukocyte count (cells/µL), median [IQR]	8800 [7315–11,598]	9950 [7938–13,502]	8510 [7150–11,250]	8130 [6570–9980]	7730 [5962–14,395]
Eosinophil count (cells/µL), median [IQR]	240 [108–728]	220 [75–502]	380 [150–890]	290 [170–830]	135 [90–1045]
**Treatment and outcomes**					
Total albendazole duration (months), median [IQR]	7 [4–13] [*n* = 63]	9.5 [6–13] [*n* = 20]	7 [4–10] [*n* = 27]	6 [3–12] [*n* = 13]	3 [3–13] [*n* = 3]
Biliary leak after intervention, *n* (%)	6 (7.0)	0 (0.0)	4 (12.9)	2 (10.5)	0 (0.0)
Follow-up duration (years), median [IQR]	2 [2–4] [*n* = 76]	2 [1–3] [*n* = 23]	2 [2–4]	3 [2–4.5] [*n* = 17]	4 [1–8] [*n* = 5]
Post-index detection of CE, *n*/*N* (%)	17/76 (22.4)	3/23 (13.0)	10/31 (32.3)	3/17 (17.6)	1/5 (20.0)

Section headings are shown in bold. Values are *n* (%) or median [IQR]; where the number with available data differs from the column total it is shown in brackets. Data are descriptive; no between-group testing was performed because treatment allocation depended on cyst stage, morphology, complications, organ involvement and local expertise. Classification hierarchy: surgery, when open or laparoscopic surgery was the initial liver-directed procedure; PAIR/Mo-CAT, when a percutaneous procedure was the initial liver-directed treatment irrespective of subsequent surgery (12 of 31 subsequently underwent surgery); albendazole alone, no liver-directed procedure and Gharbi I–III or unstaged cyst; watch-and-wait, no liver-directed procedure and Gharbi IV–V cyst. Three watch-and-wait patients received albendazole, two for concomitant extrahepatic disease. Gharbi stage, as recorded in the original imaging reports, refers to the most active hepatic cyst (largest cyst when stages were identical); approximate WHO-IWGE correspondences are Type I, CE1; Type II, CE3a; Type III, CE2 or CE3b; Type IV, CE4; Type V, CE5. PAIR and Mo-CAT were recorded as a single variable and cannot be separated. Post-index detection of CE is reported among patients with documented follow-up. Mo-CAT, modified catheterization technique; PAIR, puncture–aspiration–injection–reaspiration; CE, cystic echinococcosis; CRP, C-reactive protein; ESR, erythrocyte sedimentation rate; IQR, interquartile range.

**Table 7:** Baseline characteristics of patients with and without post-index detection of cystic echinococcosis during follow-up (patients with documented follow-up, *n* = 104).

**(a) Comparison of baseline characteristics**			
**Variable**	**Post-Index Detection (*n* = 19)**	**No Post-Index Detection (*n* = 85)**	***p* Value**
Age (years), median [IQR]	10 [7.8–12.2]	10.7 [8.3–14.2]	0.405
Male sex, *n* (%)	10 (52.6)	42 (49.4)	0.800
Rural residence, *n* (%)	8 (42.1)	43 (50.6)	0.504
Hepatic involvement, *n* (%)	17 (89.5)	59 (69.4)	0.075
Pulmonary involvement, *n* (%)	11 (57.9)	26 (30.6)	0.025
Multiorgan involvement (≥2 organs), *n* (%)	11 (57.9)	10 (11.8)	<0.001
Multiple cysts, *n* (%)	10 (52.6)	30 (35.3)	0.160
Number of cysts, median [IQR]	2 [1–4] (*n* = 18)	1 [1–2]	0.002
Largest cyst diameter (mm), median [IQR]	60 [40–90]	60 [43–82.5]	0.926
Largest cyst >5 cm, *n* (%)	11 (57.9)	57 (67.1)	0.448
Complicated cyst, *n* (%)	6 (31.6)	22 (25.9)	0.613
Surgery (any site), *n* (%)	12 (63.2)	47 (55.3)	0.532
Total albendazole duration (months), median [IQR]	13 [4–20] (*n* = 15)	7 [4–11] (*n* = 66)	0.097
Follow-up duration (years), median [IQR]	3 [2–7]	2 [1–4]	0.036
**(b) Proportion with post-index detection of CE according to baseline features**			
**Baseline Feature**	**Feature Present, Events/*n* (%)**	**Feature Absent, Events/*n* (%)**	***p* Value**
Multiorgan involvement	11/21 (52.4)	8/83 (9.6)	<0.001
Pulmonary involvement	11/37 (29.7)	8/67 (11.9)	0.025
Multiple cysts	10/40 (25.0)	9/64 (14.1)	0.160
Largest cyst >5 cm	11/68 (16.2)	8/36 (22.2)	0.448

Values are *n* (%) or median [IQR]. Chi-square or Fisher’s exact test for categorical and Mann–Whitney U test for continuous variables; univariable comparisons; analyses accounting for follow-up duration are reported in the text. Post-index detection of CE is a composite outcome (true recurrence, reactivation, reinfection, secondary dissemination or previously undetected lesion). Follow-up schedules and imaging intervals were not standardized. CE, cystic echinococcosis; IQR, interquartile range.

## Data Availability

The datasets generated and/or analyzed during the current study are available from the corresponding author on reasonable request.

## Supplementary Materials

The following supporting information can be downloaded at: https://www.mdpi.com/article/10.3390/tropicalmed11100283/s1. Figure S1: Flow of patients through the study; Table S1: Individual characteristics of the 19 patients with post-index detection of CE; Table S2: Supplementary analyses of the multivariable models (patients included in versus excluded from the complete-case analysis; sensitivity models).

## References

[1] World Health Organization (2025). WHO Guidelines for the Treatment of Patients with Cystic Echinococcosis.

[2] Liu T., Li G., Qu H., Li R., Wang X., Tang F. (2025). Trends in the global burden of cystic echinococcosis among children and adolescents from 1990 to 2021: An analysis based on the Global Burden of Disease Study 2021. PLoS Negl. Trop. Dis..

[3] Stojkovic M., Zwahlen M., Teggi A., Vutova K., Cretu C.M., Virdone R., Nicolaidou P., Cobanoglu N., Junghanss T. (2009). Treatment response of cystic echinococcosis to benzimidazoles: A systematic review. PLoS Negl. Trop. Dis..

[4] Gharbi H.A., Hassine W., Brauner M.W., Dupuch K. (1981). Ultrasound examination of the hydatic liver. Radiology.

[5] Brunetti E., Kern P., Vuitton D.A. (2010). Expert consensus for the diagnosis and treatment of cystic and alveolar echinococcosis in humans. Acta Trop..

[6] Kuehn R., Uchiumi L.J., Tamarozzi F. (2024). Treatment of uncomplicated hepatic cystic echinococcosis (hydatid disease). Cochrane Database Syst. Rev..

[7] Bakal U., Simsek S., Kazez A. (2015). Surgical and Molecular Evaluation of Pediatric Hydatid Cyst Cases in Eastern Turkey. Korean J. Parasitol..

[8] Paduraru A.A., Lupu M.A., Popoiu C.M., Stanciulescu M.C., Tirnea L., Boia E.S., Olariu T.R. (2024). Cystic Echinococcosis in Hospitalized Children from Western Romania: A 25-Year Retrospective Study. Biomedicines.

[9] Sheves A., Fuxman Y., Gazer B., Shmueli M., Van Buren J., Ben-Shimol S., Assi Z. (2023). Treatment of Cystic Echinococcosis in Children: A Single Center Experience. Pediatr. Infect. Dis. J..

[10] Yosra K., Ghada H., Bochra A., Fatma T., Said J., Riadh J. (2023). Predictive Factors of Liver Hydatid Cyst Recurrence in Children. J. Pediatr. Surg..

[11] Herrador Z., Siles-Lucas M., Aparicio P., Lopez-Velez R., Gherasim A., Garate T., Benito A. (2016). Cystic Echinococcosis Epidemiology in Spain Based on Hospitalization Records, 1997–2012. PLoS Negl. Trop. Dis..

[12] Lopez-Bernus A., Belhassen-García M., Alonso-Sardón M., Carpio-Perez A., Velasco-Tirado V., Romero-Alegria Á., Muro A., Cordero-Sánchez M., Pardo-Lledias J. (2015). Surveillance of Human Echinococcosis in Castilla-Leon (Spain) between 2000–2012. PLoS Negl. Trop. Dis..

[13] Rossi P., Tamarozzi F., Galati F., Pozio E., Akhan O., Cretu C.M., Vutova K., Siles-Lucas M., Brunetti E., Casulli A. (2016). The first meeting of the European Register of Cystic Echinococcosis (ERCE). Parasit. Vectors.

[14] Tamarozzi F., Akhan O., Cretu C.M., Vutova K., Akinci D., Chipeva R., Ciftci T., Constantin C.M., Fabiani M., Golemanov B. (2018). Prevalence of abdominal cystic echinococcosis in rural Bulgaria, Romania, and Turkey: A cross-sectional, ultrasound-based, population study from the HERACLES project. Lancet Infect. Dis..

[15] Kocaman O.H., Günendi T., Dere O., Dörterler M.E., Boleken M.E. (2022). Pulmonary Hydatid Cyst in Children: A Single-Institution Experience. Cureus.

[16] Kuzucu A., Soysal O., Ozgel M., Yologlu S. (2004). Complicated hydatid cysts of the lung: Clinical and therapeutic issues. Ann. Thorac. Surg..

[17] Mfingwana L., Goussard P., van Wyk L., Morrison J., Gie A.G., Mohammed R.A.A., Janson J.T., Wagenaar R., Ismail Z., Schubert P. (2022). Pulmonary Echinococcus in children: A descriptive study in a LMIC. Pediatr. Pulmonol..

[18] Goussard P., Eber E., Mfingwana L., Nel P., Schubert P., Janson J., Pitcher R., le Roux C. (2022). Paediatric pulmonary echinococcosis: A neglected disease. Paediatr. Respir. Rev..

[19] Mahmoudi S., Mamishi S., Banar M., Pourakbari B., Keshavarz H. (2019). Epidemiology of echinococcosis in Iran: A systematic review and meta-analysis. BMC Infect. Dis..

[20] Belhassen-Garcia M., Balboa Arregui Ó., Calabuig-Muñoz E., Carmena D., Esteban Velasco M.D.C., Fuentes Gago M., Fumado Pérez V., García Alonso J., García López Hortelano M., González Fernández L.M. (2020). Executive Summary of the Consensus Statement of the Spanish Society of Infectious Diseases and Clinical Microbiology (SEIMC), the Spanish Society of Tropical Medicine and International Health (SEMTSI), the Spanish Association of Surgeons (AEC), the Spanish Society of Pneumology and Thoracic Surgery (SEPAR), the Spanish Society of Thoracic Surgery (SECT), the Spanish Society of Vascular and Interventional Radiology (SERVEI), and the Spanish Society of Paediatric Infectious Diseases (SEIP), on the Management of Cystic Echinococcosis. Enferm. Infecc. Microbiol. Clin..

[21] Akhan O., Özbay Y., Ünal E., Karaagaoglu E., Çiftçi T.T., Akıncı D. (2025). Long-Term Results of Modified Catheterization Technique in the Treatment of CE Type 2 and 3b Liver Hydatid Cysts. Cardiovasc. Interv. Radiol..

[22] Mönnink G.L.E., Stijnis C., van Delden O.M., Spijker R., Grobusch M.P. (2021). Percutaneous Versus Surgical Interventions for Hepatic Cystic Echinococcosis: A Systematic Review and Meta-Analysis. Cardiovasc. Interv. Radiol..

[23] Manciulli T., Vola A., Mariconti M., Lissandrin R., Maestri M., Budke C.M., Tamarozzi F., Brunetti E. (2018). Shortage of Albendazole and Its Consequences for Patients with Cystic Echinococcosis Treated at a Referral Center in Italy. Am. J. Trop. Med. Hyg..

[24] Arif S.H., Malik A.A., Khaja A.R., Dass T.A., Naikoo Z.A. (2011). Role of albendazole in the management of hydatid cyst liver. Saudi J. Gastroenterol..

[25] Çay Ü., Alabaz D., Ö Gündeşlioglu Ö., Tutuş K., Çil M.K., Pehlivan U.A. (2022). Multi-organ/disseminated echinococcosis in children: Case series and review of the literature. J. Paediatr. Child Health.

[26] Ghatee M.A., Nikaein K., Taylor W.R., Karamian M., Alidadi H., Kanannejad Z., Sehatpour F., Zarei F., Pouladfar G. (2020). Environmental, climatic and host population risk factors of human cystic echinococcosis in southwest of Iran. BMC Public Health.

[27] Craig P.S., McManus D.P., Lightowlers M.W., Chabalgoity J.A., Garcia H.H., Gavidia C.M., Gilman R.H., Gonzalez A.E., Lorca M., Naquira C. (2007). Prevention and control of cystic echinococcosis. Lancet Infect. Dis..

[28] Zouari M., Jarboui O., Belhajmansour M., Hbaieb M., Issaoui A., Ben Dhaou M., Mhiri R. (2026). Predictors of Postoperative Morbidity in Pediatric Liver Hydatid Disease: Role of Comorbidities and Multisite Infection. World J. Surg..

