# Supplementary material for: Organ-Specific Clinical Patterns of Pediatric Cystic Echinococcosis: A Multicenter Cohort from an Endemic Region
Source: Trop Med Infect Dis. 2026 Oct 8;11(10):283. doi: 10.3390/tropicalmed11100283 (PMC13709892; doi:10.3390/tropicalmed11100283)
Supplement: Supplementary file 1 [file tropicalmed-11-00283-s001.zip › tropicalmed-4503484-supplementary.pdf]

Supplementary Materials

Organ-Specific Clinical Patterns of Pediatric Cystic Echinococcosis: A Multicenter Cohort from an Endemic Region

Coskun Ekemen, Asli Arslan, Melis Deniz, Sema Yildirim Arslan, Nezihe Bilge Bahçeci, Ayşe Kübra Açık, Damla Seyhanlı, Emine Cigdem Ozer, Osman Uzunlu, Ülgen Çeltik, Nursen Belet, Dicle Sener Okur, Tuğçe Tural Kara, Ilknur Caglar, Derya Dirim Erdogan, Celal Çınar, Ülküm Zafer Dökümcü, Ahmet Çelik, Metin Korkmaz, Gulhadiye Avcu and Zumrut Sahbudak Bal

Contents: Figure S1. Flow of patients through the study; Table S1. Individual characteristics of the 19 patients with post-index detection of CE; Table S2. Supplementary analyses of the multivariable models (patients included in versus excluded from the complete-case analysis; sensitivity models).

Table S1. Individual characteristics of the 19 patients with post-index detection of CE.

#	Age (y)	Sex	Rural	Organ(s) at baseline	Hepatic Gharbi stage	Largest cyst (mm)	No. of cysts	Multiple cysts	Complicated cyst	Index management	Total albendazole (months)	Follow-up (y)	Interval to detection (months)
1	2.6	M	No	Liver + Lung	III (CE2/CE3b)	58	5	Yes	No	Liver: PAIR/Mo-CAT; Procedures (any site): PAIR/Mo-CAT	21	10	18
2	4.2	F	No	Liver	II (CE3a)	40	1	No	No	Liver: Surgery; Procedures (any site): Surgery	3	4	12
3	6.2	F	No	Liver + Cranial	V (CE5)	50	2	Yes	No	Liver: Watch-and-wait; Procedures (any site): Surgery	13	9	ND
4	6.5	M	No	Liver + Lung	I (CE1)	92	2	No	No	Liver: PAIR/Mo-CAT; Procedures (any site): PAIR/Mo-CAT	20	2	4
5	7.8	M	No	Liver	III (CE2/CE3b)	39	1	No	No	Liver: Surgery; Procedures (any site): Surgery	14	1	36
6	8.1	F	No	Liver	I (CE1)	20	3	Yes	No	Liver: PAIR/Mo-CAT; Procedures (any site): PAIR/Mo-CAT	ND	4	36
7	8.2	M	No	Liver + Spleen	I (CE1)	90	2	No	No	Liver: PAIR/Mo-CAT; Procedures (any site): Surgery + PAIR/Mo-CAT	13	9	48
8	8.3	M	Yes	Lung	NA	60	1	No	Yes	Procedures (any site): Surgery	6	2	8
9	9.6	M	No	Liver	I (CE1)	125	4	Yes	No	Liver: Surgery; Procedures (any site): Surgery + PAIR/Mo-CAT	ND	10	24
10	10.0	M	Yes	Liver + Lung	I (CE1)	80	5	Yes	Yes	Liver: PAIR/Mo-CAT; Procedures (any site): PAIR/Mo-CAT	14	1	ND
11	11.0	F	No	Liver + Lung	II (CE3a)	90	5	Yes	No	Liver: Albendazole alone; Procedures (any site): Surgery	36	3	3
12	11.4	M	Yes	Liver	I (CE1)	20	1	No	No	Liver: PAIR/Mo-CAT;	ND	7	24

#	Age (y)	Sex	Rural	Organ(s) at baseline	Hepatic Gharbi stage	Largest cyst (mm)	No. of cysts	Multiple cysts	Complicated cyst	Index management	Total albendazole (months)	Follow-up (y)	Interval to detection (months)
										Procedures (any site): PAIR/Mo-CAT			
13	11.8	F	No	Liver + Lung	II (CE3a)	95	1	No	No	Liver: PAIR/Mo-CAT; Procedures (any site): Surgery + PAIR/Mo-CAT	ND	6	2
14	11.9	F	Yes	Liver + Lung	II (CE3a)	81	4	Yes	No	Liver: PAIR/Mo-CAT; Procedures (any site): Surgery + PAIR/Mo-CAT	3	2	ND
15	12.2	F	No	Lung	NA	20	nan	No	Yes	Procedures (any site): None (albendazole only)	39	3	36
16	13.3	M	Yes	Liver + Lung	II (CE3a)	50	3	Yes	Yes	Liver: PAIR/Mo-CAT; Procedures (any site): Surgery + PAIR/Mo-CAT	3	6	ND
17	13.8	F	Yes	Liver + Lung	III (CE2/CE3b)	40	4	Yes	Yes	Liver: Albendazole alone; Procedures (any site): Surgery	13	2	13
18	14.8	F	Yes	Liver	I (CE1)	120	1	No	No	Liver: PAIR/Mo-CAT; Procedures (any site): Surgery + PAIR/Mo-CAT	7	3	ND
19	16.9	M	Yes	Liver + Lung	I (CE1)	61	2	Yes	Yes	Liver: Albendazole alone; Procedures (any site): None (albendazole only)	4	1	5

ND, not documented in the medical record (not "not treated"); NA, not applicable (no hepatic involvement). Index hepatic management follows the hierarchy defined in the Methods; "procedures" lists surgery and/or percutaneous treatment received at any site. According to current WHO terminology, true recurrence, reactivation, reinfection, secondary dissemination and newly detected lesions are distinct entities; because the anatomical site of the subsequent lesion and its relation to the treated site were not consistently recorded, these could not be differentiated and post-index detection is a composite outcome that is not interpreted as a recurrence endpoint. The interval to detection is the median among the 14 event cases with a documented date of detection; in the remaining 5 cases the date of the imaging study on which the lesion was first noted was not recorded. Cases with and without timing data were similar in follow-up duration (median 3.5 [2–7.5] vs 3 [1.5–7.5] years), pulmonary involvement (8/14 vs 3/5) and multiorgan involvement (7/14 vs 4/5). No survival analysis was performed because event timing was incomplete and follow-up duration was recorded in whole years.

Table S2. Supplementary analyses of the multivariable models (patients included in versus excluded from the complete-case analysis; sensitivity models.

(a) Patients included in (n = 83) versus excluded from (n = 36) the complete-case multivariable analysis (exclusion was determined solely by missing CRP)

Variable	Included (n = 83)	Excluded (n = 36)	p value
Age (years), median [IQR]	10 [7.9–13.3] (n=83)	11.2 [8.6–14.1] (n=36)	0.149
Male sex, n (%)	43 (51.8)	17 (47.2)	0.646
Rural residence, n (%)	44 (53.0)	14 (38.9)	0.157
Pulmonary involvement, n (%)	37 (44.6)	6 (16.7)	0.004
Multiorgan involvement, n (%)	23 (27.7)	1 (2.8)	0.002
Fever, n (%)	29 (34.9)	3 (8.3)	0.003
Complicated cyst, n (%)	29 (34.9)	3 (8.3)	0.003
Largest cyst >5 cm, n (%)	55 (66.3)	21 (58.3)	0.408
Hospitalization required, n (%)	73 (88.0)	25 (69.4)	0.015

(b) Multivariable logistic regression for pulmonary involvement: primary model and sensitivity models

Variable	Adjusted OR	95% CI	p value
Primary model: rural residence, fever, CRP (n = 83; pulmonary cases = 37; χ^2 = 24.16, p <0.001; Nagelkerke R^2 = 0.338)			
Rural residence	1.71	0.59–4.90	0.322
Fever	5.58	1.85–16.81	0.002
CRP (per 10 mg/L increase)	1.13	1.01–1.27	0.038
Model A: primary model + male sex (n = 83; pulmonary cases = 37; χ^2 = 25.19, p <0.001; Nagelkerke R^2 = 0.35)			
Rural residence	1.63	0.56–4.73	0.370
Fever	5.02	1.63–15.45	0.005
CRP (per 10 mg/L increase)	1.12	0.99–1.26	0.063
Male sex	1.73	0.60–4.94	0.308
Model B: primary model + male sex + age (n = 83; pulmonary cases = 37; χ^2 = 25.35, p <0.001; Nagelkerke R^2 = 0.352)			
Rural residence	1.71	0.57–5.11	0.338
Fever	4.86	1.56–15.11	0.006
CRP (per 10 mg/L increase)	1.12	1.00–1.26	0.059
Male sex	1.64	0.56–4.84	0.367
Age (per year)	0.97	0.85–1.11	0.687
Model C: primary model + center (coordinating vs other centers) (n = 83; pulmonary cases = 37; χ^2 = 24.23, p <0.001; Nagelkerke R^2 = 0.339)			
Rural residence	1.62	0.53–4.95	0.396

Variable	Adjusted OR	95% CI	p value
Fever	5.45	1.79–16.59	0.003
CRP (per 10 mg/L increase)	1.13	1.01–1.28	0.037
Coordinating center	1.17	0.39–3.53	0.781
Model D: rural residence and fever (without CRP; all patients) (n = 119; pulmonary cases = 43; χ^2 = 21.66, p <0.001; Nagelkerke R^2 = 0.228)			
Rural residence	1.80	0.78–4.15	0.171
Fever	6.10	2.45–15.17	<0.001

(a) Values are n (%) or median [IQR]; chi-square or Fisher's exact test for categorical and Mann–Whitney U test for continuous variables. All 36 excluded patients came from three centers in which CRP was not routinely recorded. (b) Candidate variables were those associated with pulmonary involvement in univariable analysis (male sex, rural residence, fever, multiorgan involvement, complicated cyst, CRP, leukocyte count, IHA titer) plus age as an a priori confounder; respiratory symptoms, hospitalization and surgery were not considered (consequences of pulmonary localization), multiorgan involvement was not considered (definitionally linked to the outcome), complicated cyst status was not considered (a manifestation of pulmonary localization), leukocyte count was omitted in favour of the collinear CRP and IHA titer was omitted because of missingness. Variables were entered simultaneously (Enter method); models A–C are complete-case analyses of the 83 patients with CRP available and model D includes all 119 patients. The primary model after multiple imputation of missing CRP values is shown in Table 4 of the main text. Complicated cyst, infection, rupture or peri-cystic infection documented on imaging or at operation. CI, confidence interval; CRP, C-reactive protein; IHA, indirect hemagglutination assay; IQR, interquartile range; OR, odds ratio.

Children aged 0–18 years with a definitive diagnosis of cystic echinococcosis, September 2013 – September 2023, six tertiary centers in Turkey

↓

Entered into the study database: n = 119 (Center 1 [coordinating center], 39; Center 2, 32; Center 3, 19; Center 4, 10; Center 5, 10; Center 6, 9). Only patients meeting the definitive-diagnosis criteria were entered; the number of records screened but not meeting the criteria was not logged.

↓

Excluded after entry: 0

↓

Analyzed for baseline characteristics, organ involvement and univariable comparisons: n = 119

↓

Complete data for the multivariable model (rural residence, fever, CRP): n = 83 | excluded for missing CRP: n = 36

↓

Hepatic involvement: n = 86 (albendazole alone 30; PAIR/Mo-CAT 31; surgery 19; watch-and-wait 6) | Pulmonary involvement: n = 43

↓

Evaluable for the follow-up outcome (post-treatment follow-up documented): n = 104 | not evaluable (no documented follow-up): n = 15

↓

Post-index detection of CE: n = 19 (date of detection documented: 14)

Figure S1. Flow of patients through the study.
